# A Rare Case of Argatroban-Induced Anaphylaxis in a Patient With Intermediate-High Risk Pulmonary Embolism

**DOI:** 10.7759/cureus.61129

**Published:** 2024-05-26

**Authors:** Kishen G Bulsara, Humail Patel, Aaron Goldstein, Merlin Mathew

**Affiliations:** 1 Internal Medicine, Northwell Health, New Hyde Park, USA

**Keywords:** heparin-induced thrombocytopenia (hit), drug-induced anaphylaxis, heparin drip, argatroban, saddle pulmonary embolism

## Abstract

Heparin-induced thrombocytopenia is a rare and potentially devastating complication of heparin therapy. Patients with an absolute indication for anticoagulation, such as those with significant pulmonary embolism, must be switched to a different anticoagulant, such as argatroban, a direct thrombin inhibitor. We report a case of anaphylaxis to argatroban in a patient who was initially on heparin for intermediate-high risk pulmonary embolism but developed suspected type II heparin-induced thrombocytopenia. This case highlights the significance of recognizing and treating anaphylactic reactions and the diagnostic challenges associated with heparin-induced thrombocytopenia.

## Introduction

Pulmonary embolism (PE) is characterized by the obstruction of a pulmonary artery, most often caused by the propagation of a thrombus from a lower extremity vein. The management of PE is dictated by risk stratification and ranges widely from emergent intervention for high-risk PE to oral anticoagulation for low-risk PE. Treatment of intermediate-risk PE, the remainder of cases, is variable and often requires hospitalization for close monitoring given the high risk of clinical decompensation [1].

The mainstay of treatment of acute PE is anticoagulation to prevent clot propagation while allowing for physiologic thrombolysis to occur. There are several possible options for anticoagulation, including direct oral anticoagulants (apixaban, rivaroxaban, dabigatran, or edoxaban), vitamin K antagonists (warfarin), and for most hospitalized patients, intravenous unfractionated heparin infusion or subcutaneous enoxaparin [2]. A rare, life-threatening complication of heparin therapy is type II heparin-induced thrombocytopenia (HIT), described as an acute drop in platelets after heparin exposure due to the development of platelet factor 4 (PF4) autoantibodies. Type II HIT can be seen in patients with prior heparin exposure and can cause a hypercoagulable state resulting in venous and arterial thromboses [3]. The workup of HIT includes laboratory evaluation for PF4 IgG antibodies (highly sensitive) and the serotonin release assay (highly specific) [3]. Once HIT is suspected, heparin should be discontinued, and patients should be transitioned to an alternative intravenous anticoagulant, such as argatroban or bivalirudin (direct thrombin inhibitors) [3,4].

To date, there have been no reported cases of allergic reactions linked to intravenous argatroban although per the manufacturer, signs and symptoms of a possible reaction include cough, shortness of breath, and skin flushing [5]. Herein, we report a rare case of anaphylaxis after the initiation of argatroban for suspected HIT in a patient admitted with intermediate-high risk PE.

## Case presentation

Clinical summary

A 32-year-old female with a past medical history of left lower extremity deep venous thrombosis (DVT) and saddle PE with mild right ventricular dysfunction (diagnosed three months prior to admission), obesity, and active tobacco use presented with worsening shortness of breath on exertion, mid-sternal chest pain, and left lower extremity pain with swelling. The patient initially presented three months prior with similar symptoms and was discharged on subcutaneous enoxaparin twice daily for anticoagulation. The patient was unable to refill her prescription after the first month and was without anticoagulation for two months prior to admission.

On presentation, vital signs were notable for sinus tachycardia (heart rate: 138) with normal blood pressure and oxygen saturation. Physical examination was notable for mild respiratory distress with tachypnea, tachycardia, and marked left calf swelling with tenderness to palpation. Laboratory data revealed an elevated serum brain natriuretic peptide (BNP) to 2636 pg/mL, elevated lactate to 2.2 mmol/L, and normal high-sensitivity troponin T, coagulation profile, blood counts, and electrolytes.

**Figure 1:**
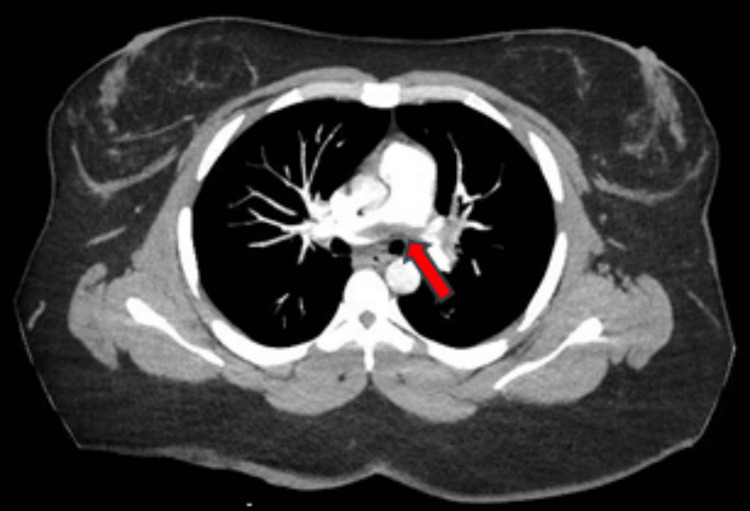
Initial CT angiogram from three months prior to admission showing extensive bilateral saddle pulmonary embolism (red arrow).

**Figure 2:**
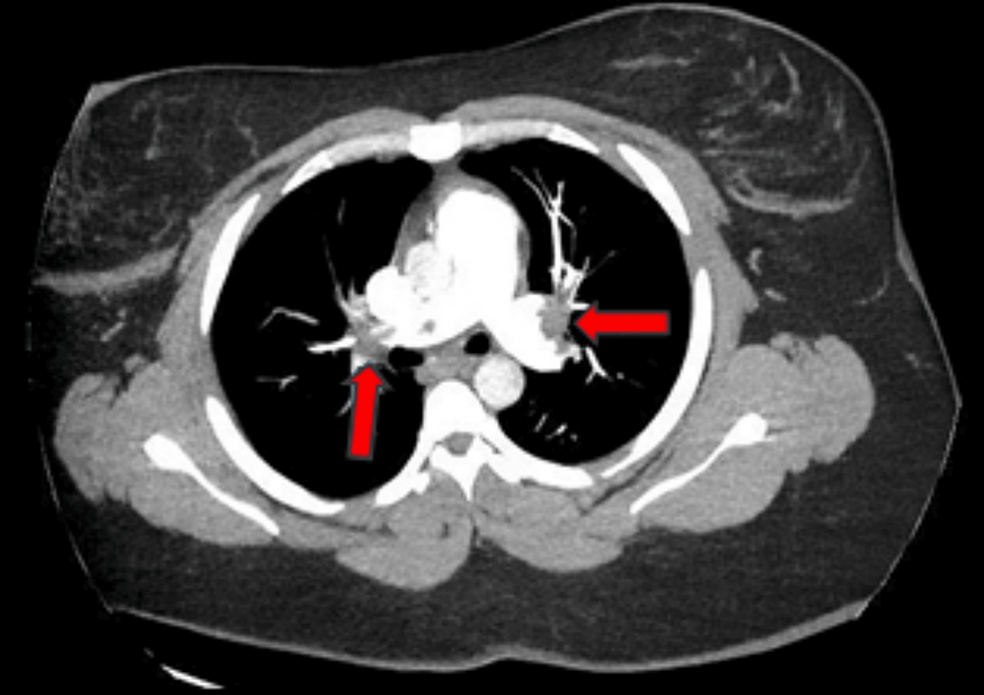
Repeat CT angiogram showing decreased saddle pulmonary embolism but significant intraluminal defects in multiple lobes (red arrows).

**Figure 3:**
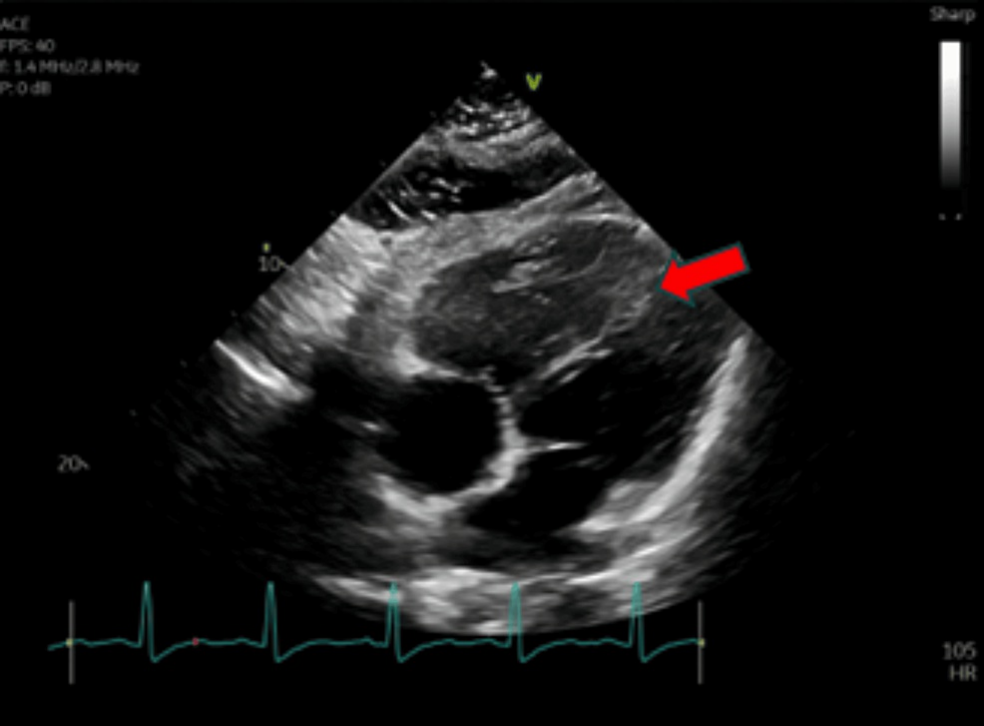
Transthoracic echocardiogram showing interventricular septal flattening, concerning for elevated right-sided pressures (red arrow).

CT angiography of the chest showed decreased saddle PE compared to initial CT angiography from three months prior (Figure 1) but was notable for significant intraluminal defects in all lobes (Figure 2). Duplex ultrasound confirmed above- and below-the-knee DVTs in the patient’s left lower extremity. Transthoracic echocardiography was notable for mildly decreased left ventricular ejection fraction (45-50%), interventricular septal flattening, severe pulmonary hypertension (pulmonary artery systolic pressure of 83 mmHg), and moderate tricuspid regurgitation (Figure 3). EKG was notable for sinus tachycardia with a heart rate of 113, right-sided deviation, and T-wave inversions in leads III and V1-V4, suggesting right heart strain. Given the right heart strain, elevated BNP, and a simplified pulmonary embolism severity index (sPESI) score > 0, this patient’s PE was classified as intermediate-high risk. She was evaluated by the cardiac intensive care unit but was not deemed a candidate for admission.

Treatment and hospital course

The patient was started on an unfractionated heparin infusion for anticoagulation. Three days into admission, her platelet count was noted to drop acutely from 266k to 107k. Given the drop in platelets >50% with platelet nadir ≥20 and no apparent other cause for thrombocytopenia, the patient’s 4T score for HIT was 4 (intermediate probability). A PF4 IgG antibody test and serotonin release assay were subsequently sent to evaluate for HIT with the former returning positive within several hours, suggesting possible type II HIT. Unfractionated heparin was immediately discontinued, and the patient was switched to an argatroban infusion. Approximately five minutes after the initiation of argatroban, the patient reported acute-onset shortness of breath, chest and throat tightness, pruritus, and diffuse hives. Vital signs during this time were notable for sinus tachycardia (heart rate of 120s) and hypoxia with oxygen saturation of 91% on two liters of oxygen. Due to concern for anaphylaxis, the argatroban drip was immediately discontinued and she received intramuscular epinephrine and diphenhydramine with marked improvement in symptoms. Hematology was consulted for further anticoagulation recommendations and recommended transitioning to fondaparinux.

Outcome

The patient underwent right heart catheterization, which revealed elevated right atrial pressure (11 mmHg) and mild pulmonary hypertension (pulmonary artery systolic pressure of 42 mmHg) responsive to pulmonary vasodilator therapy. She was initiated on sildenafil 10 mg three times daily for pulmonary hypertension treatment. By this point, the patient’s serotonin release assay returned negative and her platelet counts had normalized, and it was concluded that the patient likely never had true HIT. Nonetheless, she was evaluated by the allergy-immunology team for the anaphylactic reaction to argatroban and was recommended for outpatient allergy testing in six weeks. She was ultimately discharged home with subcutaneous enoxaparin for anticoagulation with close follow-up with a primary care physician, pulmonology, vascular cardiology, allergy-immunology, and hematology.

## Discussion

Most hospitalized patients with intermediate-risk pulmonary embolism are on anticoagulation, often with unfractionated heparin [1,2]. Providers managing any patient being treated with heparin should be cognizant of HIT as a potential life-threatening complication. We present a rare case of anaphylaxis in the setting of intravenous argatroban use in a patient being treated for intermediate-high risk PE. Per the manufacturer, argatroban may cause possible allergic reactions presenting as cough, dyspnea, and a drop in blood pressure [5] but no documented cases of severe hypersensitivity or anaphylaxis have been reported in the literature to date. Our patient had rapid onset of shortness of breath, chest and throat tightness, skin involvement (pruritis and hives), and mild hypoxia, meeting the criteria for anaphylaxis per the European Academy of Allergy and Clinical Immunology [6]. Fortunately, she had significant improvement after the administration of intramuscular epinephrine and diphenhydramine, underscoring the importance of prompt recognition and treatment of anaphylaxis due to its life-threatening nature [6]. During her hospitalization, she was seen by the allergy-immunology team who recommended formal allergy testing as an outpatient in three to six weeks as testing too early increases the false-negative rate due to depleted histamine stores [7].

Interestingly, our patient’s workup for HIT was ultimately negative given the negative serotonin-release assay. It should be noted that PF4 IgG antibody testing is highly sensitive (>99%) with rapid results but has low specificity (30-70%) and a high false-positive rate. On the other hand, serotonin release assays are highly specific (>95%) but have lower sensitivities (56-100%) and typically take several days to result in our institution [8]. Due to our patient’s marked drop in platelets, intermediate 4T score, and positive PF4 IgG antibodies, there was clinical suspicion for type II HIT and the decision was made to switch from unfractionated heparin to argatroban. Argatroban and bivalirudin are approved intravenous anticoagulants in suspected and confirmed HIT [3,4]. Alternatives to these therapies, including fondaparinux and direct oral anticoagulants, have also been studied and shown to have similar rates of platelet recovery, safety profiles (i.e., major bleeding), and risk of new or worsening thromboembolism [4,9]. After our patient’s anaphylactic reaction to argatroban, she was transitioned to fondaparinux for continued treatment of her PE before switching back to twice-daily subcutaneous enoxaparin on discharge.

## Conclusions

HIT is a life-threatening complication of heparin therapy that can cause significant morbidity and mortality. The management of suspected and confirmed HIT involves switching to alternative and less commonly used anticoagulants, such as argatroban and bivalirudin. Though it has yet to be reported in the literature, argatroban therapy may result in an anaphylactic reaction, necessitating immediate discontinuation and intramuscular epinephrine treatment. Further studies are required to elucidate the specific mechanisms of argatroban-induced anaphylaxis.
